# Stability estimation through multivariate approach among solasodine-rich lines of *Solanum khasianum* (C.B. Clarke): an important industrial plant

**DOI:** 10.3389/fpls.2023.1143778

**Published:** 2023-05-11

**Authors:** Twahira Begum, Sunita Munda, Tanmita Gupta, Roktim Gogoi, Vikash Kumar Choubey, Sanjoy K. Chanda, Himangshu Lekhak, G. N. Sastry, Mohan Lal

**Affiliations:** ^1^ Agrotechnology and Rural Development Division, Council of Scientific and Industrial Research (CSIR)-North East Institute of Science and Technology, Jorhat, Assam, India; ^2^ Advanced Computation and Data Sciences Division, Council of Scientific and Industrial Research (CSIR)-North East Institute of Science and Technology, Jorhat, Assam, India

**Keywords:** anova, AMMI, GGE biplot, MTSI, Shukla’s variance, solasodine, *Solanum khasianum*

## Abstract

*Solanum khasianum* is a medicinally important plant that is a source of steroidal alkaloids ‘solasodine.’ It has various industrial applications, including oral contraceptives and other pharmaceutical uses. The present study was based on 186 germplasm of *S. khasianum*, which were analyzed for the stability of economically important traits like solasodine content and fruit yield. The collected germplasm was planted during *Kharif* 2018, 2019, and 2020 in RCBD with three replications at the experimental farm of CSIR-NEIST, Jorhat, Assam, India. A multivariate approach for stability analysis was applied to identify stable germplasm of *S. khasianum* for economically important traits. The germplasm was analyzed for additive main effects and multiplicative interaction (AMMI), GGE biplot, multi-trait stability index, and Shukla’s variance which were evaluated for three environments. The AMMI ANOVA revealed significant GE interaction for all the studied traits. The stable and high-yielding germplasm was identified from the AMMI biplot, GGE biplot, Shukla’s variance value, and MTSI plot analysis. Lines no. 90, 85, 70, 107, and 62 were identified as highly stable fruit yielders while, lines no. 1, 146, and 68 were identified as stable high solasodine lines. However, considering both traits, i.e., high fruit yield and solasodine content, MTSI analysis was performed which showed that lines 1, 85, 70,155, 71, 114, 65, 86, 62, 116, 32, and 182 could be used in a breeding program. Thus, this identified germplasm can be considered for further varietal development and could be used in a breeding program. The findings of the present study would be beneficial for the *S. khasianum* breeding program.

## 1 Introduction

The Solanaceae family with worldwide distribution comprises 90 genera, containing more than 3000 species. The Solanaceae family’s largest genus is Solanum with more than 2000 species (Kaunda and Zhang, 2019). *Solanum khasianum* (C.B. Clarke), also called *Solanum aculeatissimum* Jacq., is a member of the Solanaceae family and is cultivated in China, Bhutan, and the Indian states of Assam, Arunachal Pradesh, Meghalaya, Sikkim, Manipur, Orissa, Nilgiri Hill, and West Bengal (Gogoi et al., 2021). Alkaloids, flavonoids, phenols, and numerous other phytoconstituents found in *S. khasianum* are recognized to have the ability to reduce inflammation, blood sugar levels, and oxidative stress (Gupta, 2019). The potential glycoalkaloids reported to be present in *S. khasianum* include solasodine, solamargine, solanine, and solasonine (Srivastava et al., 2016; Kaunda and Zhang, 2019). Among the Solanum plants screened for solasodine production, *S. khasianum* was found to possess the maximum solasodine content (Weissenberg, 2001; Begum et al., 2022; Lal et al., 2022a). It is widely known for its range of therapeutic applications. Solasodine was found to possess many pharmacological properties like anti-inflammatory, antioxidant, antinociceptive, antimicrobial, cytotoxic, anti-obesity, antiandrogenic, and hepatoprotective properties (Srivastava et al., 2016; Kumar et al., 2019; Gogoi et al., 2021). Moreover, previous reports suggested that the berries possess anti-diabetic, anti-inflammatory, anti-cholinesterase, anticancer, and antibacterial properties (Rosangkima and Jagetia, 2015; Gogoi et al., 2021; Pavani and Shasthree, 2021). As an ethnomedicine, the plant has been used for treating diseases like whooping cough, bronchitis, smallpox, trachoma, snake bites, rheumatism, and tooth and skin infections (Schmelzer and Gurib, 2008; Chirumamilla et al., 2021; Lal et al., 2022a).

*Solanum khasianum* is a sturdy, branched shrub with a height of approximately 0.75 to 1.5 m. The stem and leaves bear spines on both its surfaces with the shape of ovate to lobate. It possesses hermaphrodite flowers and abundant berries and turns yellowish upon maturity (Begum et al., 2022; Lal et al., 2022a). The cultivation area of *S. khasianum* is more than 4000 ha in India across the states of Assam, Meghalaya, Tripura, Manipur, Maharashtra, and Karnataka (Gogoi et al., 2021). *S. khasianum* is a storehouse of solasodine, the steroidal alkaloid which has high industrial and medicinal significance (Sanwal et al., 2016; Gogoi et al., 2021). It is the starting base for synthesizing steroid hormones, such as cortisone and oral contraceptives (Begum et al., 2022). Solasodine is a key intermediate and substrate in steroidal drugs and sex hormone production (Sanwal et al., 2016; Lal et al., 2022a). It is used in steroidal hormone production for commercial use in oral pills or injections (Sinani and Eltayeb, 2017; Lal et al., 2022a). It, thus, plays a crucial role in human birth control purposes. The valuable part of the plant is the berries which contain the source of solasodine that can be extracted from ripened berries. The maximum content of solasodine is produced when the berries have ripened and turned yellow in color. The seeds are mucilage in nature, small in size, and are the primary source of propagation (Begum et al., 2022). Increased consumption of this medicine and reckless harvesting of the plant from the wild as a raw material source has led to the depletion of genetic diversity and habitat loss (Canter et al., 2005; Julsing et al., 2007). Due to its rapid growth and low initial culture cost, it is one of the best sources of affordable raw materials for industrially significant solasodine extraction. Therefore, it would be very advantageous to identify and include plant breeding for this neglected plant species.

As for the definition of stability, a genotype that can consistently produce consistent performance regardless of any change in its environmental conditions can be termed a stable genotype. In plant breeding, the stability of yield traits is a significant aspect. However, with ever-changing fluctuations in environmental conditions, the determination of yield factor is challenging. The phenotypic expression of a genotype is a controlled result of both genotypic constitutions of the genotype and the environment. Hence, the genotype may show inconsistent performance under varying environmental conditions. A stable genotype with superior yield in varying environments is considered the mostly desired genotype. This compels the necessary study of the genotype × environment to identify stable genotypes with desirable traits.

A stable genotype is one which produces a stable yield in an environment (Annicchiarico, 2002; Munda et al., 2020). The performance of a genotype in multi-environments is difficult to study when the genotype-environment interaction is stronger (Alwala et al., 2010). The selection of a stable genotype is difficult due to the interaction between genotype × environments (Gupta et al., 2015). The genotypic response to an environment is multivariate; hence, the determination of stability using parametric and nonparametric statistical analysis which is univariate would not be very effective. There are many multivariate models available for the analysis of stability. Among the various multivariate model, additive main effects and multiplicative interaction (AMMI) method and ‘GGE biplot’ are widely used (Lal et al., 2022b). The AMMI model is an amalgamation of the analysis of variance and the principal component analysis. It efficiently illustrates the adaptive responses of genotypes to the environment by providing details on the main and interaction effects along with a biplot. The main effects of G × E are assessed using variance analysis (main additive effects) while the G × E interaction is estimated using the principal analysis components (multiplicative interactions). The GGE biplot was developed to represent graphically the G × E interaction and genotype main effects. Shukla’s variance analysis is effective to partition the total G × E interaction for the estimation of the variance attribution for each of the genotypes in an unbiased manner. For the simultaneous estimation of stability and identification of genotypes with multiple superior traits, the MTSI is useful for exploiting wide adaptability. The plant breeding program is aimed at the identification of superior yielding lines due to varying genotypes’ performance and varying environments. Thus, the results obtained are either for the identification of genotypes with high-yielding traits or different environment-adapted genotypes. However, with the multivariate approach for stability analysis across multilocation, both the issues of varying genotypic and environmental interactions can be addressed. The present stability analysis was verified by AMMI, GGE, MTSI, and Shukla’s variance methods. High fruit-yielding germplasm with high solasodine content, which shows a consistent performance across different locations of NE India, is the major selection criteria for the identification of elite lines.

So far, none of the stability studies in *S. khasianum* are available in the public domain. Hence the present study was conducted for the first time to assess the performance of large numbers of germplasm of *S. khasianum* with high solasodine content.

## 2 Materials and methods

### 2.1 Materials and methods

Initially, 273 germplasm of *S. khasianum* were collected from different regions of NE India. Among them, 186 high solasodine content-rich (>0.8%) lines were identified and used in the present study (Begum et al., 2022). The identified 186 germplasm were planted for three years in *Kharif* in 2018, 2019, and 2020 at the experimental farm of CSIR-North East Institute of Science and Technology (NEIST), Jorhat, Assam, India, and were evaluated for three years. The plant specimens were identified by the plant breeder of CSIR-NEIST, Jorhat, India, and the prepared herbarium was deposited in the departmental record with identification ID: RRLJ-SK-1 to RRLJ-SK-186.

### 2.2 Morphological characters recorded

The morphological data were recorded for the three years of study. The data included plant spread (cm), plant height (cm), leaf width (cm), leaf length (cm), fruit diameter (cm), number of fruits/plants, fresh fruit weight per plant (kg), and days to maturity. Along with the morphological characters, the solasodine content (%) was also recorded for each year (**Table S1**). All the data were recorded for ten randomly selected healthy plants for each germplasm.

### 2.3 Solasodine extraction and quantification

The dried ripe berries of *S. khasianum* were used for the estimation of solasodine. Solasodine extraction and quantification were done as per the method suggested by Begum et al. (2022).

### 2.4 Statistical analysis

R software was used for performing all the statistical analyses. The mean-multi-environment trial analysis package in R software was used in the study (Olivoto et al., 2019). The analysis of variance (ANOVA), AMMI, multi-trait stability index (MTSI), GGE, and Shukla’s variance were used to analyze the three-year data of *Solanum khasianum* for economic traits like solasodine content and fresh fruit weight per plant.

## 3 Results

The evaluation of germplasm is pivotal to elite germplasm; therefore, the identified or selected germplasm needs to be further assessed for its stability and consistent performance in varied environments. The present study focuses on the identification of stable germplasm of *S. khasianum* for economically important traits. The pooled data for three years were analyzed with ANOVA and various stability analyses (AMMI, GGE, MTSI, and Shukla’s variance). The coefficient of variation is a significant parameter for the comparison between traits based on phenotypic variation. Concerning the coefficient of variation, a higher variation was found in the fresh fruit weight per plant followed by plant height while a moderate coefficient of variation was observed for solasodine content and days to maturity (**Table 1**).

**Table 1:** The analysis of variance on the three-year data of *Solanum khasianum* germplasm that was used in the study.

Source		PH		PS		LL		LW		NOF		FD		FWP		SOL		DOM	
	DF	SS%	MS	SS%	MS	SS%	MS	SS%	MS	SS%	MS	SS%	MS	SS%	MS	SS%	MS	SS%	MS
ENV	2	376989.419	188494.710	8049.256	4024.628	19.915	9.957	0.932	0.466	2172.553	1086.277	27.026	13.513	0.021	0.011	0.007	0.004	2588.649	1294.324
REP (ENV)	6	4285.538	714.256	1788.864	298.144	0.339	0.057	0.477	0.080	2842.380	473.730	12.929	2.155	0.026	0.004	0.085	0.014	624.781	104.130
GEN	185	416682.056	2252.335	1693809.673	9155.728	793.179	4.287	1642.942	8.881	1235319.272	6677.401	393.950	2.129	1.739	0.009	10.606	0.057	132502.973	716.232
GEN × ENV	370	569760.358	1539.893	171910.078	464.622	374.612	1.012	136.330	0.368	13090.780	35.380	68.407	0.185	1.668	0.005	2.167	0.006	61248.018	165.535
Residuals	1110	985714.462	888.031	117275.136	105.653	171.167	0.154	311.590	0.281	112908.953	101.720	127.211	0.115	4.892	0.004	8.601	0.008	144715.885	130.375
Total	2043	376989.419	188494.710	8049.256	4024.628	19.915	9.957	0.932	0.466	2172.553	1086.277	27.026	13.513	0.021	0.011	0.007	0.004	2588.649	1294.324
CV (%)		34.014		10.659		5.746		8.542		16.457		5.046		38.526		9.470		6.361	

ENV, environment; REP, replication; GEN, genotype; DF, degree of freedom; MS, mean sum of squares; PH,plant height; PS= plant spread; LL, leaf length; LW, leaf width; NOF, number of fruit/plant; FD, fruit diameter; FWP, fresh fruit weight/plant; SOL, solasodine content; DOM= days to maturity; CV, coefficient of variation.

**Figure 1:**
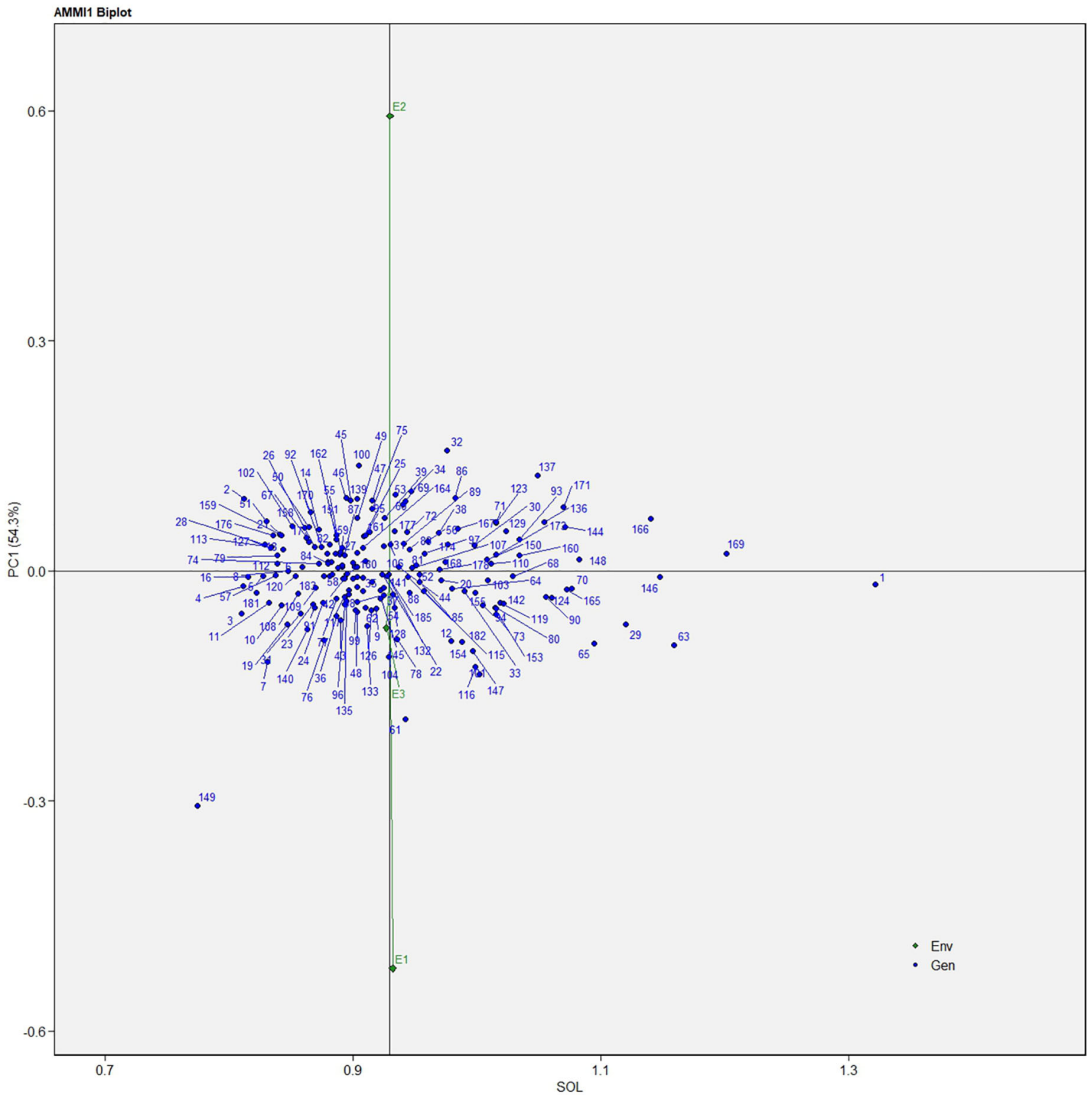
AMMI biplot model 1 for solasodine content in 186 germplasm of *S. khasianum*.

**Figure 2:**
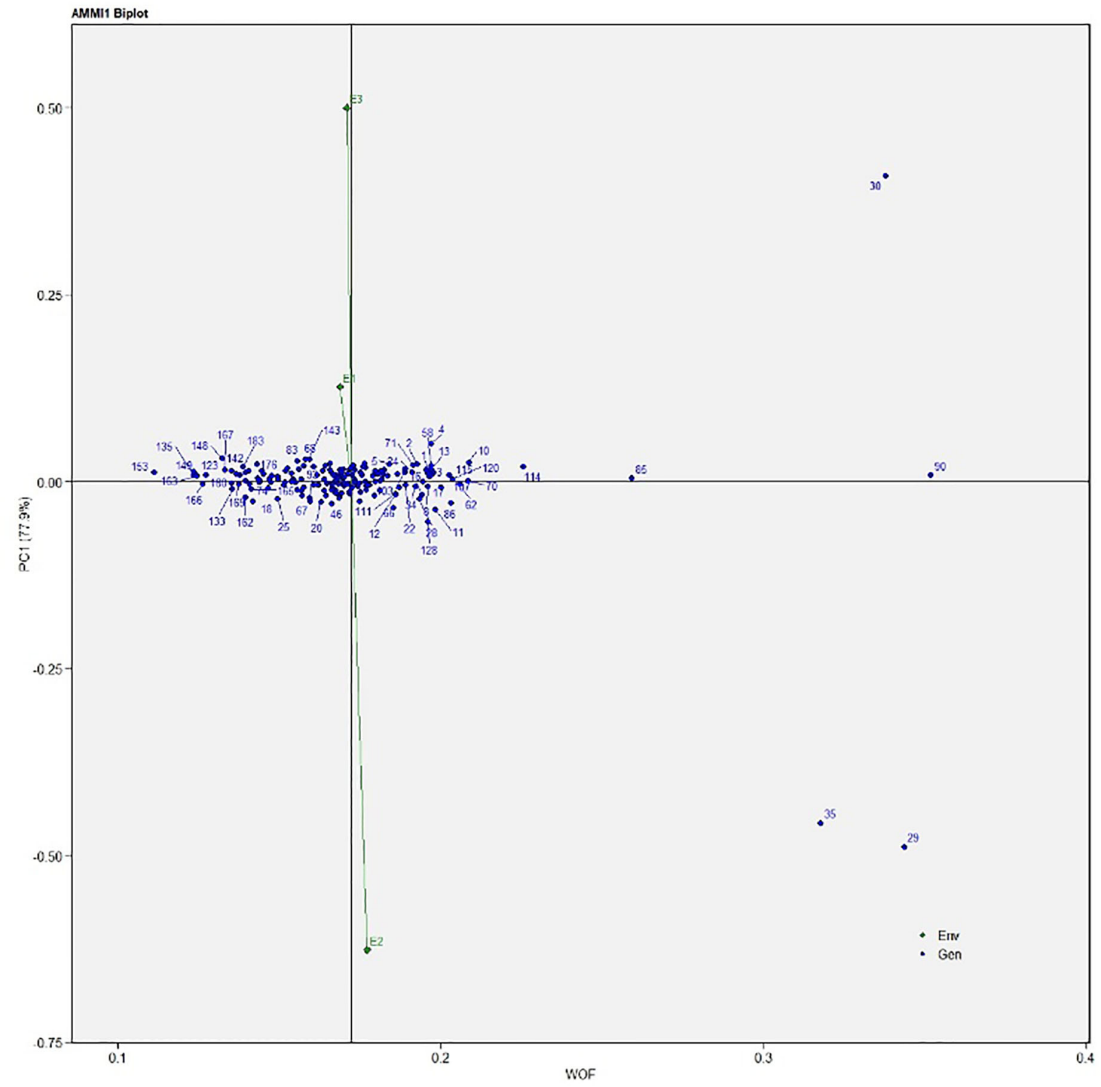
AMMI biplot model 1 for the weight of fruit in 186 germplasm of *S. khasianum*.

**Figure 3:**
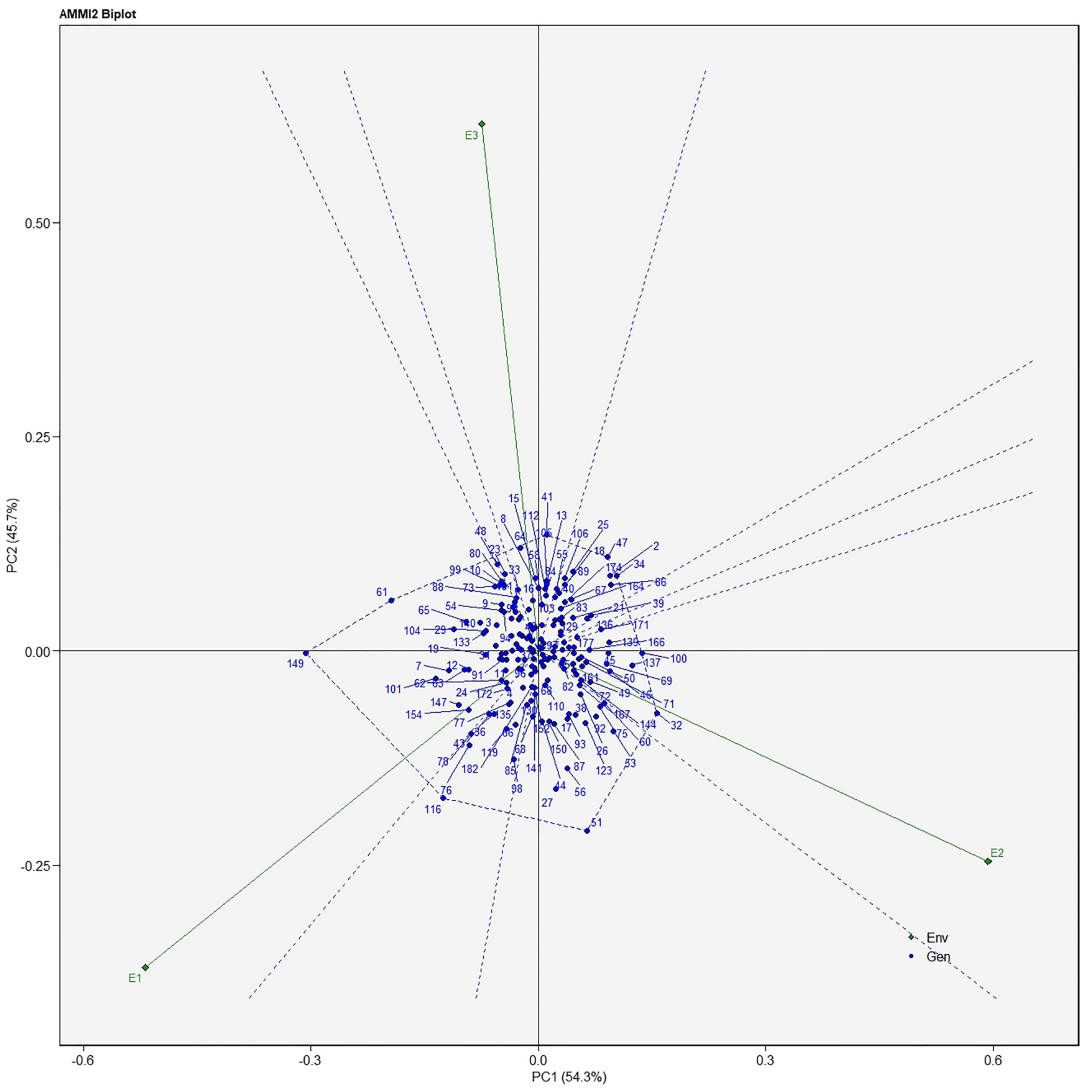
AMMI biplot model 2 for solasodine content in 186 germplasm of *S. khasianum*.

The AMMI analysis of variance (ANOVA) revealed that the environmental effect was significant for all the traits while the genotypic effect was significant for plant height, fruit diameter, fresh fruit weight per plant, solasodine content, and days to maturity. Significant G × E interaction was observed for all the studied traits indicating divergent performances across the different environments. Thus, a precise selection of germplasm with elite traits is essential for a breeding program. The interaction effect was analyzed from the principal component analysis (IPCA) of AMMI ANOVA from which G × E interaction sum of squares percentage value of 48.660 and 16.955 was recorded for fresh fruit weight per plant and solasodine content, respectively (**Table 2**). A more precise analysis for the selection of germplasm with elite traits from the genotype-environment interaction was identified through the AMMI biplot. The genotype-environment interaction for yield is an important analysis in which the less interacting genotypes are closer to the center of the AMMI biplot and considered more stable than their counterpart for that region. In contrast, the far-centered genotypes have individual stability with each environment which indicates the high-performing germplasm for each environment. The AMMI was performed for the economically important traits *i.e.*, solasodine content and fresh fruit weight per plant for which AMMI biplots were constructed. From the AMMI results, it was found that lines no. 1, 146, 68, 178, 52, 107, and 44 were among the high yielders and were stable for solasodine content (**Figure 1**). Lines no. 90, 85, 70, 107, and 62 were considered stable and high yielders for fresh fruit weight per plant (**Figure 2**). The individual competence of the genotypes in the studied environments was evaluated using the AMMI2 model in which the first two principal components (Factor 1 and Factor 2) were used to generate the plot. In the AMMI2 biplot for solasodine content, line no. 116 was considered a high yielder in environment one, line no. 51 and 32 in environment two, and line no. 105 in environment three, respectively (**Figure 3**).

**Table 2:** The AMMI Analysis of variance on the three-year data of *Solanum khasianum* germplasm that was used in the study.

A																
Source	DF	PH			PS			LL			LW			NOF		
		SS	MS	%	SS	MS	%	SS	MS	%	SS	MS	%	SS	MS	%
ENV	2	376989.4	188494.7	27.650***	8049.256	4024.628	0.430***	19.915	9.957	1.677***	0.932	0.466	0.052***	2172.553	1086.277	0.174*
REP (ENV)	6	4285.538	714.2563	0.314*	1788.864	298.144	0.095***	0.339	0.057	0.029*	0.477	0.080	0.027*	2842.380	473.730	0.227***
GEN	185	416682.1	2252.335	30.561***	1693809.673	9155.728	90.396	793.179	4.287	66.782	1642.942	8.881	92.290	1235319.272	6677.401	98.780
GEN × ENV	370	569760.4	1539.893	41.789***	171910.078	464.622	9.175***	374.612	1.012	31.541***	136.330	0.368	7.658***	13090.780	35.380	1.047*
PC1	186	462109.9	2484.462	33.893	169330.977	910.382	9.037	370.031	1.989	31.155	133.316	0.717	7.489	9711.842	52.214	0.777*
PC2	184	107650.4	585.0567	7.896*	2579.101	14.017	0.138*	4.581	0.025	0.386	3.014	0.016	0.169	3378.938	18.364	0.270*
Residuals	1110	985714.5	888.031	72.297	117275.136	105.653	6.259	171.167	0.154	14.412	311.590	0.281	17.503	112908.953	101.720	9.029
Total	2043	2923192	1430.833	27.650	2164743.084	1059.590	0.430	1733.825	0.849	1.677	2228.601	1.091	0.052	1379424.719	675.196	0.174
B																
Source	DF	FD			FWP			SOL			DOM					
		SS	MS	%	SS	MS	%	SS	MS	%	SS		MS		%	
ENV	2	27.026	13.513	5.522**	0.021	0.011	0.615**	0.0071	0.0035	0.055*	2588.649		1294.324		1.318***	
REP (ENV)	6	12.929	2.155	2.642***	0.026	0.004	0.762**	0.0846	0.0141	0.662**	624.781		104.130		0.318*	
GEN	185	393.950	2.129	80.499***	1.739	0.009	50.725***	10.6063	0.0573	82.989***	132502.973		716.232		67.487***	
GEN × ENV	370	68.407	0.185	13.978***	1.668	0.005	48.660**	2.1670	0.0059	16.955*	61248.018		165.535		31.195***	
PC1	186	59.108	0.318	12.078	1.300	0.007	37.907	1.1767	0.0063	9.207*	34686.809		186.488		17.667***	
PC2	184	9.299	0.051	1.900	0.369	0.002	10.753	0.9903	0.0054	7.748*	26561.209		144.354		13.528**	
Residuals	1110	127.211	0.115	25.994	4.892	0.004	142.686	8.6013	0.0077	67.301	144715.885		130.375		73.707	
Total	2043	697.930	0.342	5.522	10.016	0.005	0.615	1110	8.601	0.055	402928.324		197.224		1.318	

ENV, environment; REP, replication; GEN, genotype; PC, Principal component; DF, degree of freedom; MS, mean sum of squares; FD, Fruit diameter; FWP, fresh fruit weight/plant; SOL, Solasodine content; DOM, Days to maturity; CV, coefficient of variation, %, explained %; ***, significant at p <0.005; **, significant at p < 0.01; *, significant at p < 0.05.

**Figure 4:**
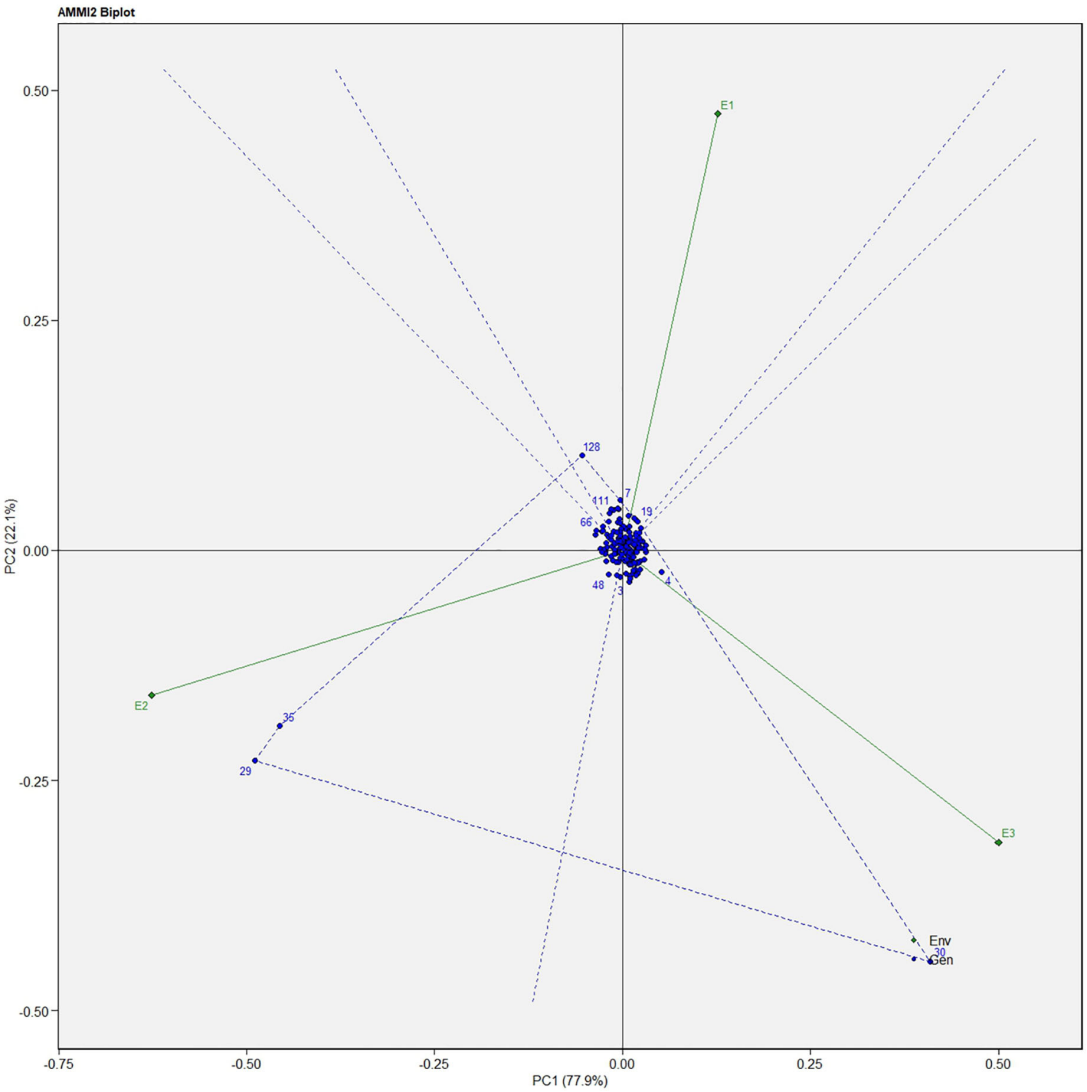
AMMI biplot model 2 for the weight of fruit in 186 germplasm of *S. khasianum*.

Similarly, for the fresh fruit weight per plant, line no. 128 and 7 were considered stable in environment one, lines no. 29 and 35 in environment two, and line no. 30 in environment three, respectively (**Figure 4**). The above-identified genotypes showed the best performance in the studied environments and were found to be highly competent.

**Figure 5:**
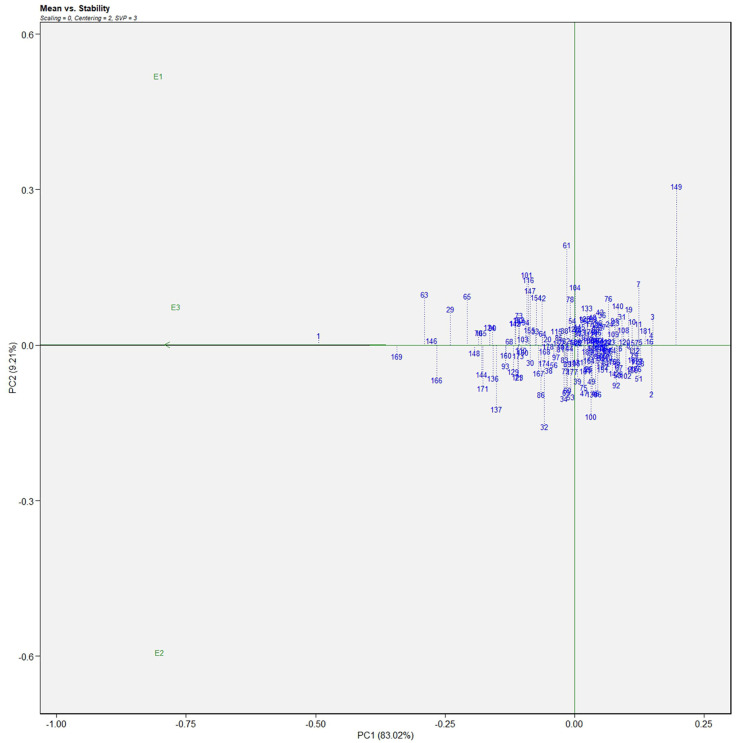
Average environment coordination views of the GGE-biplot based on environment-focused scaling for the mean performance and stability 186 germplasm of *S. khasianum* for solasodine content.

**Figure 6:**
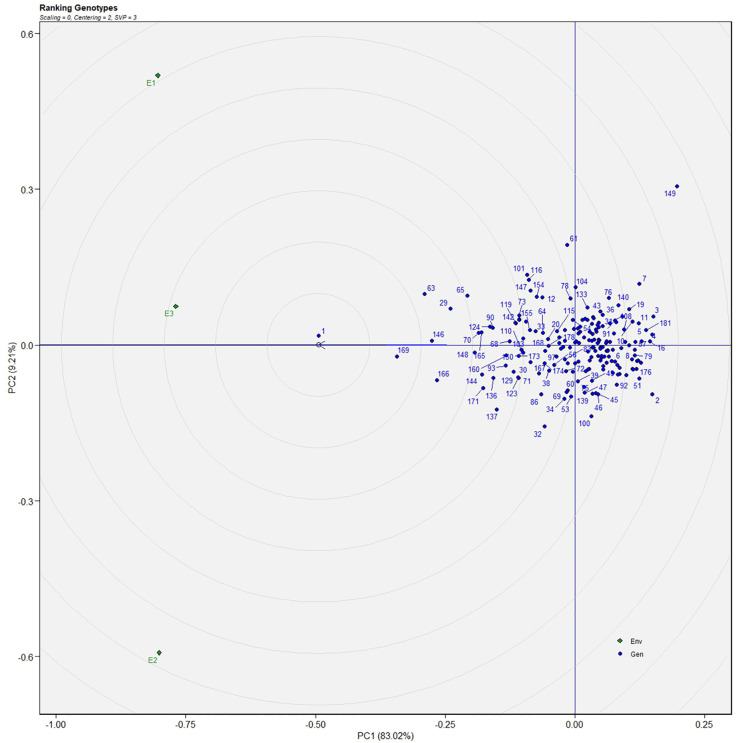
GGE-biplot based on genotype and environment-focused scaling for the comparison of genotype and environments for solasodine content.

**Figure 7:**
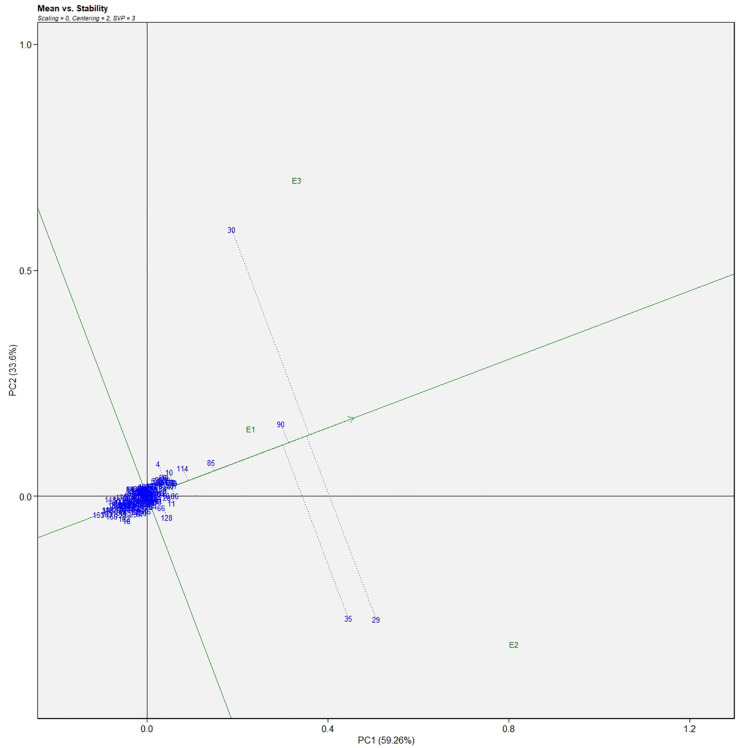
Average environment coordination views of the GGE-biplot based on environment-focused scaling for the mean performance and stability 186 germplasm of *S. khasianum* for the weight of fruit.

**Figure 8:**
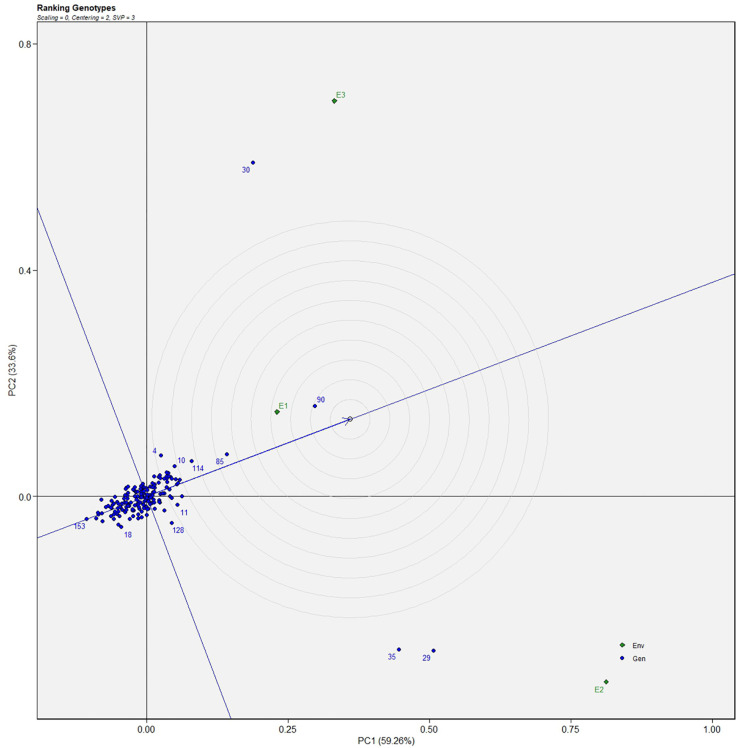
GGE-biplot based on genotype and environment-focused scaling for the comparison of genotype and environments for the weight of fruit.

The genotype in a studied environment that possesses the highest mean performance and absolute stability can be considered the ideal genotype. Such a genotype possessing a superior trait with zero-G × E interaction is ideal. It should be described as the one possessing the most excellent vector length from genotype markers to the origin of the biplot. In the GGE biplot, lines no. 149, 61, and 32 were the most unstable lines. Their vector lengths were longer compared to the other genotypes. Whereas lines no.1, 169, and 146 had high solasodine content and were found to be stable genotypes since their vector lengths were short (**Figures 5**, **6**). While considering the trait of the fresh fruit weight per plant from the GGE biplot, genotypes 29, 30, and 35 had higher weights of fresh fruit/plant than average but were unstable as they exhibited longer vector lengths. Lines no. 90, 85, and 114 had high fresh fruit weight per plant and exhibited short vector lengths which characterize a stable genotype (**Figures 7**, **8**). The discriminating ability is directly proportional to the vector length. Environments E2 and E3 had longer vectors than E1 for the trait fresh fruit weight per plant (**Figure 8**). While, for the trait solasodine content, E1 and E2 had longer vectors than E3 (**Figure 6**). Thus, E2 and E3 were the best environments to exhibit germplasm differentiation for fresh fruit yield per plant while environment E1 and E2 were the most discriminating environment for solasodine content.

**Figure 9:**
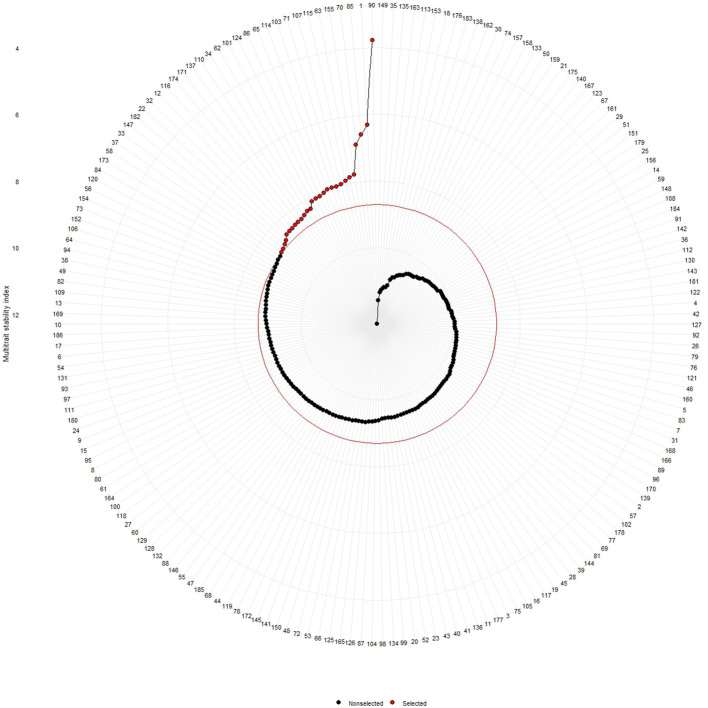
Multi trait stability index (MTSI) of 186 germplasm of *S. khasianum* based on the traits of solasodine content and weight of fruit.

Shukla’s variance was studied for genotype performance across the environments according to how the germplasm with minimum values is intended to be more stable. On the basis of our present results, lines no. 153, 135, 149, 163, 166, and 123 were considered stable for fresh fruit weight per plant while lines no. 149, 3, 4, 2, 16, 181, 5, and 28 were considered stable for solasodine content (**Table 3**). The MTSI study revealed that the mean of the selected germplasm (Xs) was higher than the mean of the original (Xo) for fresh fruit weight per plant and solasodine content. The selection gain (SG) was positive for both traits with SG% of 7.022 and 7.610 for the traits of fresh fruit weight per plant and solasodine content, respectively. The selection differential (SD) was favorable for both traits and heritability of 0.520 and 0.873 for fresh fruit weight per plant and solasodine content, respectively (**Table 4**). As per the MTSI analysis, lines no. 90, 1, 85, 70, 155, 63, 115, 107, 71, 103, 114, 65, 86, 124, 101, 62, 34, 110, 137, 171, 174, 116, 12, 32, 22, 182, 147, and 33 were considered as stable germplasm (**Figure 9**). Based on the multivariate and univariate approaches used in the study, it can be concluded that lines 90, 85, 107, and 62 were the elite lines with a high yield and stable nature. The study of stability is an important aspect in the identification of stable genotypes with elite traits. The elite germplasm in the study refers to the stable germplasm with a high fruit yield and solasodine content. The study of *S. khasianum* is significant for commercially cultivating the industrially important solasodine-rich plant.

**Table 3:** Shukla’s variance on the three-year data for solasodine content and weight of fruit for *Solanum khasianum* germplasm that was used in the study.

Shukla’s variance for solasodine content						Shukla’s variance for fresh fruit weight per plant					
GEN	Y	GEN	Y	GEN	Y	GEN	Y	GEN	Y	GEN	Y
1	1.321	63	1.158	125	0.894	1	0.197	63	0.169	125	0.172
2	0.812	64	0.98	126	0.914	2	0.192	64	0.174	126	0.169
3	0.81	65	1.094	127	0.833	3	0.196	65	0.173	127	0.176
4	0.811	66	0.896	128	0.932	4	0.197	66	0.185	128	0.195
5	0.827	67	0.862	129	1.023	5	0.184	67	0.159	129	0.153
6	0.858	68	1.028	130	0.907	6	0.196	68	0.158	130	0.149
7	0.831	69	0.942	131	0.924	7	0.177	69	0.155	131	0.179
8	0.847	70	1.076	132	0.927	8	0.195	70	0.208	132	0.167
9	0.918	71	1.015	133	0.911	9	0.179	71	0.191	133	0.135
10	0.842	72	0.943	134	0.895	10	0.208	72	0.166	134	0.171
11	0.832	73	1.015	135	0.894	11	0.198	73	0.161	135	0.123
12	0.978	74	0.872	136	1.054	12	0.186	74	0.140	136	0.144
13	0.882	75	0.915	137	1.048	13	0.197	75	0.167	137	0.169
14	0.872	76	0.876	138	0.891	14	0.154	76	0.160	138	0.136
15	0.883	77	0.886	139	0.903	15	0.181	77	0.164	139	0.161
16	0.815	78	0.935	140	0.863	16	0.194	78	0.166	140	0.146
17	0.864	79	0.838	141	0.928	17	0.200	79	0.171	141	0.169
18	0.843	80	1.015	142	1.021	18	0.141	80	0.155	142	0.135
19	0.846	81	0.951	143	0.903	19	0.182	81	0.153	143	0.159
20	0.971	82	0.881	144	1.071	20	0.162	82	0.195	144	0.139
21	0.842	83	0.945	145	0.922	21	0.151	83	0.155	145	0.168
22	0.923	84	0.88	146	1.147	22	0.189	84	0.194	146	0.140
23	0.867	85	0.953	147	0.996	23	0.174	85	0.259	147	0.171
24	0.875	86	0.982	148	1.082	24	0.191	86	0.203	148	0.132
25	0.908	87	0.893	149	0.774	25	0.149	87	0.171	149	0.123
26	0.861	88	0.945	150	1.007	26	0.162	88	0.166	150	0.155
27	0.903	89	0.941	151	0.886	27	0.173	89	0.157	151	0.151
28	0.828	90	1.055	152	0.953	28	0.193	90	0.351	152	0.177
29	1.12	91	0.868	153	1.014	29	0.343	91	0.157	153	0.111
30	0.997	92	0.865	154	0.987	30	0.337	92	0.163	154	0.171
31	0.857	93	1.034	155	0.998	31	0.172	93	0.159	155	0.196
32	0.975	94	1.004	156	0.9	32	0.176	94	0.163	156	0.147
33	0.99	95	0.91	157	0.885	33	0.169	95	0.180	157	0.139
34	0.946	96	0.893	158	0.868	34	0.192	96	0.165	158	0.143
35	0.915	97	0.957	159	0.841	35	0.317	97	0.166	159	0.151
36	0.886	98	0.924	160	1.034	36	0.154	98	0.168	160	0.135
37	0.924	99	0.902	161	0.913	37	0.181	99	0.172	161	0.143
38	0.968	100	0.904	162	0.886	38	0.175	100	0.181	162	0.139
39	0.925	101	1.002	163	0.922	39	0.164	101	0.187	163	0.124
40	0.878	102	0.851	164	0.907	40	0.175	102	0.179	164	0.177
41	0.9	103	1.008	165	1.072	41	0.171	103	0.188	165	0.141
42	0.893	104	0.928	166	1.14	42	0.156	104	0.167	166	0.126
43	0.89	105	0.88	167	0.984	43	0.169	105	0.176	167	0.133
44	0.936	106	0.93	168	0.974	44	0.169	106	0.178	168	0.146
45	0.897	107	0.947	169	1.201	45	0.167	107	0.205	169	0.137
46	0.894	108	0.855	170	0.874	46	0.166	108	0.164	170	0.169
47	0.915	109	0.87	171	1.07	47	0.170	109	0.197	171	0.166
48	0.903	110	1.011	172	0.903	48	0.179	110	0.173	172	0.171
49	0.903	111	0.901	173	1.015	49	0.183	111	0.186	173	0.168
50	0.864	112	0.838	174	0.976	50	0.147	112	0.168	174	0.179
51	0.83	113	0.834	175	0.888	51	0.163	113	0.137	175	0.145
52	0.887	114	0.892	176	0.835	52	0.172	114	0.225	176	0.143
53	0.934	115	0.956	177	0.933	53	0.166	115	0.202	177	0.165
54	0.933	116	0.998	178	0.97	54	0.169	116	0.175	178	0.152
55	0.89	117	0.893	179	0.907	55	0.181	117	0.172	179	0.143
56	0.96	118	0.891	180	0.91	56	0.175	118	0.182	180	0.178
57	0.837	119	1.018	181	0.822	57	0.177	119	0.157	181	0.171
58	0.903	120	0.853	182	0.944	58	0.197	120	0.203	182	0.188
59	0.891	121	0.903	183	0.876	59	0.157	121	0.160	183	0.138
60	0.94	122	0.881	184	0.887	60	0.172	122	0.156	184	0.149
61	0.942	123	1.015	185	0.927	61	0.168	123	0.127	185	0.167
62	0.91	124	1.06	186	0.896	62	0.203	124	0.174	186	0.186

GEN, genotype; Y, variance response.

**Table 4:** Estimates of selection differential, selection gain, and heritability based on MTSI for 186 *S. khasianum* germplasm across three environments.

VAR	Factor	Xo	Xs	SD	SD %	h^2^	SG	SG %
FWP	FA 1	0.172324	0.195579	0.023256	13.49529	0.520354	0.012101	7.022332
SOL	FA 1	0.929534	1.010556	0.081022	8.716357	0.873092	0.070739	7.610179

Xo, Overall mean of genotypes; Xs, Mean of the selected genotypes; SD, Selection differential; SG, Selection gain or impact; h^2^, heritability; FWP, fresh fruit weight/plant; SOL, Solasodine content.

## 4 Discussion

The assessment of the different landraces worldwide is crucial for identifying and preserving genetically rich resources. The various landraces of different crops have been the storehouse for significant desirable traits. These are widely used in various breeding programs, which can be exploited to increase genetic variability. Hashemi et al. (2018) stated that different crop genotypes exhibit variable responses under different environmental conditions; hence, to reduce the influence of the environment on the genotypes, the compatibility and stability of the genotype need to be studied for multiple years at the multilocation. The most preferable genotype from the breeding point of view is the one exhibiting stability along with a significantly positive main effect, or in other terms, which has broad adaptability (Lal et al., 2022c; Munda et al., 2023). So far, no stability analysis has been studied to date for *S. khasianum*; hence, the results were considered with previous reports of the Solanaceae family.

A study on *Solanum melongena* from Iran was performed by Khankahdani et al. (2022) on fifteen germplasm for stability analysis by using the AMMI model. The AMMI biplot revealed that genotype 7 (AM5) followed by genotypes 12 (Y7), 5 (AM7), and 13 (SA15) were identified and ranked for their stability against yield factor since they revealed the lowest genotype × environment interaction. The least stable genotypes were the ones that exhibited the highest level of genotype × environment interaction, and the most unstable identified genotypes were genotypes 1 (Y), 8 (AM4), and 19 (GHE12), respectively. Similar results were obtained in our study on the basis of which germplasm with commercially important traits and stability were identified for future breeding programs.

A similar study by Bagheri et al. (2016) reported the AMMI stability assay where 22 germplasm of the long eggplant were studied for three years. A report on the stability analysis in tobacco by the AMMI model has been previously reported by Sadeghi and Samizadeh (2011). The AMMI model was also used to study stability in general along with individual stability in different studied environments for production yield in numerous other crops like cotton (Damavandi-Kamali et al., 2011), barley (Koocheki et al., 2012), bread wheat (Bigonah, 2012), Soybean (Soltan Mohamadi et al., 2017), and durum wheat (Sadeghzadeh et al., 2018). Scavo et al. (2023) assessed six genotypes of *Solanum tuberosum* L. for AMMI and GGE. The study revealed the Salad Blue genotype as the most suitable genotype for total phenolic content, dry matter, FRAP, and vitamin C throughout eight studied environments due to its above-average mean values and stability. Another study by Das et al. (2021) was conducted on 21 potato cultivars in the eastern sub-Himalayan plains of West Bengal, India, to assess their suitability, stability, and yield factors. The study revealed ‘Kufri Pukhraj’ and ‘Kufri Chipsona-3’ as higher yielder cultivars along with ‘Kufri Surya’ as the heat-resistant cultivar. However, two cultivars, namely, ‘Kufri Khyati’and ‘HPS II/67’ were identified as stable for eastern Himalayan conditions. A study on stability analysis for nine genotypes of Elite Irish Potato (*Solanum tuberosum* L.) was conducted in western Ethiopia. The study revealed CIP384321.30 as the stable genotype for the region on the basis of the mean tuber yield, regression slope identified, and GGE biplot (Fufa and Fufa, 2021). Similar conclusions were drawn from our present study in which stable germplasm was identified across the studied environments along with individual stable germplasm for each environment with the which-won-where pattern approach.

A study on the African tomato landraces was reported by Tembe et al. (2018) in which the morphological traits were assessed. The study revealed that significant variations among the studied samples can be useful as a potential source of genetic diversity for the crop improvement of tomatoes. Similar previous findings on other vegetable crops have also been reported by different workers in tomato (Manzano et al., 2015; Sousaraei et al., 2021), lettuce (Missio et al., 2018), and eggplant fields (Taher et al., 2017; Gramazio et al., 2019), identifying superior genetic resources. Tonk et al. (2011) studied the GGE biplot analysis in maize, revealing that the G16 hybrid was found to have high suitability to the tested environments for grain yield. A study was conducted on 10 genotypes of potatoes in different environments for AMMI by Almohammedi et al. (2019). The percentage of variation of 98.44%, 0.24%, and 1.31% was recorded for genotypes, environments, and GEI, respectively, which revealed that genotypes contributed more towards variation than the other parameters. As per the definition by Bernardo (2002), when the progeny of a genotype shows consistent results for the traits of interest, the genotype is regarded as possessing a good breeding value. Based on our study, lines no.1, 90, 146, 68, 85, 107, 70, and 62 were identified as superior stable germplasm. Germplasm no. 90, 85, 70, 107, and 62 were identified as high yielding and stable for fresh fruit weight per plant. While germplasm no. 1, 146, and 68 were identified for high solasodine stable lines. Thus, this identified germplasm can be considered for further large-scale cultivation on a commercial level or can be used in breeding programs.

## 5 Conclusions

The natural source of solasodine from *S. khasianum* is an important medicinal plant species. The identification of high fruit-yielding and solasodine-rich germplasm is significant for the commercial success of the industrially important solasodine. The multivariate stability analysis is a novel approach that provides an easy way to select high-performance and stable lines in *S. khasianum* that performs better under varied environmental conditions. On the basis of the present study, lines no. 90, 85, 70, 107, and 62 were identified as stable and high-yielding fruit yields per plant. Further, lines no. 1, 146, and 68 were identified as stable and high-solasodine lines. MTSI analysis revealed lines 1, 85, 70, 155, 71, 114, 65, 86, 62, 116, 32, and 182 as stable for both high yielders, high fruit yields, and high solasodine lines. The large-scale cultivation of this germplasm can prove to be a cheap natural source of steroidal alkaloid solasodine. Moreover, the AMMI, GGE, MTSI, and Shukla’s variance models were found to be effective plant breeding models for the evaluation of stability which would maximize the utilization of resources thereby making a significant contribution to the sustainability of breeding programs. The identified genotypes of the study can be utilized for commercial cultivation and can be useful in future breeding programs.

## Data availability statement

The original contributions presented in the study are included in the article/**Supplementary Material**. Further inquiries can be directed to the corresponding author.

## Author contributions

TB: wrote the original draft, methodology, and edited the manuscript; SM: software analysis; TG: wrote the original draft; RG: editing; VKC: data curation; SC: formal analysis and visualization; HL: data curation and formal analysis; GNS: editing and supervision; ML: conceptualization, methodology, editing, validation, and supervision. All authors contributed to the article and approved the submitted version.
