# Supplementary material for: Stability estimation through multivariate approach among solasodine-rich lines of Solanum khasianum (C.B. Clarke): an important industrial plant
Source: Front Plant Sci. 2023 May 11;14:1143778. doi: 10.3389/fpls.2023.1143778 (PMC10211244; doi:10.3389/fpls.2023.1143778)
Supplement: Supplementary file 1 [file Table_1.docx]

Stability estimation through multivariate approach among solasodine rich lines of *Solanum khasianum* (C.B. Clarke): An important industrial plant

Table S1: Average morphological and quality data of 186 germplasms of *Solanum khasianum* used in the present three year study

| Pedigree | Plant height(cm) | Plant spread(cm) | Leaf length(cm) | Leaf width(cm) | No. of fruits/plant | Fruit diameter(cm) | Fresh fruit weight /plant (kg) | Days to maturity | Solasodine % |
| --- | --- | --- | --- | --- | --- | --- | --- | --- | --- |
| SK-1 | 85.333 | 88.667 | 7.678 | 7.433 | 60.667 | 6.611 | 0.186 | 158.889 | 0.863 |
| SK-2 | 78.222 | 78.778 | 7.444 | 6.533 | 62.778 | 6.467 | 0.208 | 167.889 | 0.917 |
| SK-3 | 82.667 | 77.444 | 7.089 | 6.311 | 71.111 | 7.456 | 0.186 | 167.000 | 1.260 |
| SK-4 | 93.000 | 109.889 | 5.611 | 6.411 | 83.111 | 7.744 | 0.211 | 158.778 | 0.820 |
| SK-5 | 87.556 | 74.667 | 7.189 | 6.378 | 65.889 | 6.878 | 0.195 | 177.778 | 0.861 |
| SK-6 | 116.111 | 130.000 | 5.978 | 4.233 | 69.667 | 6.167 | 0.196 | 190.889 | 0.859 |
| SK-7 | 80.556 | 83.333 | 6.911 | 6.433 | 46.111 | 6.500 | 0.352 | 178.889 | 0.813 |
| SK-8 | 87.222 | 82.000 | 6.278 | 6.122 | 43.556 | 6.300 | 0.212 | 169.222 | 0.867 |
| SK-9 | 85.889 | 80.111 | 7.433 | 6.667 | 47.111 | 6.622 | 0.175 | 166.444 | 0.993 |
| SK-10 | 86.000 | 80.667 | 6.911 | 6.678 | 35.667 | 6.289 | 0.224 | 168.444 | 0.851 |
| SK-11 | 70.333 | 59.333 | 7.256 | 6.511 | 27.111 | 6.522 | 0.184 | 158.333 | 0.829 |
| SK-12 | 88.667 | 84.222 | 7.533 | 7.256 | 27.111 | 6.844 | 0.155 | 174.889 | 1.059 |
| SK-13 | 97.444 | 78.111 | 6.878 | 6.467 | 37.222 | 6.700 | 0.202 | 182.889 | 0.817 |
| SK-14 | 95.778 | 74.000 | 7.189 | 6.900 | 41.333 | 6.956 | 0.154 | 174.778 | 0.917 |
| SK-15 | 77.556 | 79.333 | 7.322 | 6.622 | 72.556 | 6.644 | 0.165 | 173.556 | 0.950 |
| SK-16 | 109.778 | 114.333 | 6.511 | 5.211 | 40.778 | 7.767 | 0.183 | 187.667 | 0.828 |
| SK-17 | 69.444 | 70.333 | 6.867 | 6.078 | 23.111 | 6.989 | 0.181 | 183.444 | 0.929 |
| SK-18 | 84.556 | 92.111 | 7.500 | 7.078 | 45.333 | 6.722 | 0.122 | 184.556 | 0.889 |
| SK-19 | 80.444 | 80.333 | 7.267 | 6.456 | 44.556 | 6.511 | 0.176 | 178.333 | 0.873 |
| SK-20 | 92.333 | 84.333 | 6.933 | 6.556 | 35.778 | 6.611 | 0.145 | 196.333 | 1.039 |
| SK-21 | 85.444 | 84.889 | 6.711 | 5.667 | 81.000 | 6.789 | 0.152 | 187.333 | 0.819 |
| SK-22 | 81.889 | 79.444 | 7.322 | 7.144 | 30.778 | 6.689 | 0.166 | 175.556 | 0.891 |
| SK-23 | 95.333 | 88.000 | 6.822 | 4.478 | 34.222 | 5.833 | 0.173 | 175.444 | 0.917 |
| SK-24 | 86.889 | 78.000 | 7.333 | 6.656 | 46.333 | 6.644 | 0.195 | 183.333 | 0.857 |
| SK-25 | 93.000 | 115.667 | 5.644 | 6.700 | 58.889 | 7.289 | 0.142 | 162.333 | 0.856 |
| SK-26 | 84.444 | 85.000 | 7.378 | 6.444 | 65.667 | 6.900 | 0.162 | 152.556 | 0.853 |
| SK-27 | 87.222 | 124.000 | 5.589 | 6.200 | 73.889 | 6.844 | 0.173 | 166.222 | 0.809 |
| SK-28 | 94.556 | 105.333 | 7.767 | 7.367 | 56.333 | 7.378 | 0.182 | 182.111 | 0.816 |
| SK-29 | 80.333 | 81.889 | 7.689 | 6.944 | 47.778 | 7.800 | 0.154 | 171.889 | 1.224 |
| SK-30 | 86.778 | 83.556 | 6.922 | 6.644 | 30.444 | 6.567 | 0.174 | 161.667 | 1.037 |
| SK-31 | 75.000 | 74.556 | 6.856 | 6.456 | 37.778 | 6.411 | 0.152 | 160.111 | 0.864 |
| SK-32 | 75.889 | 78.444 | 7.211 | 6.678 | 49.444 | 6.556 | 0.173 | 160.333 | 0.974 |
| SK-33 | 74.778 | 165.444 | 7.622 | 6.800 | 32.111 | 6.144 | 0.157 | 154.778 | 1.129 |
| SK-34 | 92.778 | 225.556 | 6.656 | 5.389 | 26.111 | 7.244 | 0.169 | 168.111 | 1.049 |
| SK-35 | 84.333 | 294.556 | 7.278 | 6.689 | 115.444 | 7.844 | 0.151 | 183.444 | 0.829 |
| SK-36 | 77.778 | 84.111 | 6.878 | 6.800 | 44.556 | 6.733 | 0.154 | 175.778 | 0.830 |
| SK-37 | 102.333 | 100.222 | 7.700 | 7.356 | 45.667 | 6.389 | 0.169 | 172.667 | 1.031 |
| SK-38 | 79.000 | 78.444 | 7.178 | 6.078 | 32.556 | 6.789 | 20.046 | 187.556 | 0.882 |
| SK-39 | 85.778 | 81.556 | 7.467 | 6.733 | 47.444 | 6.622 | 0.149 | 183.556 | 0.820 |
| SK-40 | 85.778 | 82.444 | 6.611 | 4.744 | 52.000 | 6.856 | 0.165 | 184.778 | 0.872 |
| SK-41 | 91.111 | 90.444 | 6.344 | 6.011 | 77.889 | 6.667 | 0.171 | 178.000 | 0.816 |
| SK-42 | 77.444 | 85.667 | 6.711 | 6.478 | 51.111 | 6.756 | 0.143 | 194.444 | 0.833 |
| SK-43 | 82.444 | 74.333 | 7.200 | 6.544 | 79.556 | 6.489 | 0.153 | 186.222 | 0.808 |
| SK-44 | 93.444 | 129.889 | 5.233 | 6.578 | 45.889 | 6.811 | 0.152 | 179.889 | 0.940 |
| SK-45 | 88.667 | 80.444 | 6.767 | 6.622 | 63.556 | 6.811 | 0.172 | 178.778 | 0.892 |
| SK-46 | 86.667 | 88.778 | 7.611 | 7.056 | 57.444 | 6.756 | 0.140 | 181.778 | 0.821 |
| SK-47 | 80.667 | 88.778 | 6.489 | 5.889 | 34.778 | 7.289 | 0.178 | 183.667 | 0.858 |
| SK-48 | 80.778 | 84.889 | 7.478 | 7.011 | 42.000 | 6.678 | 0.156 | 173.444 | 0.812 |
| SK-49 | 90.667 | 92.333 | 7.956 | 7.478 | 60.444 | 6.467 | 0.166 | 167.222 | 0.842 |
| SK-50 | 87.667 | 74.222 | 7.456 | 6.644 | 101.444 | 6.667 | 0.148 | 164.111 | 0.811 |
| SK-51 | 82.444 | 81.889 | 7.022 | 6.200 | 56.778 | 6.833 | 0.149 | 167.222 | 0.948 |
| SK-52 | 77.444 | 80.222 | 7.356 | 6.167 | 66.333 | 7.033 | 0.172 | 156.778 | 0.853 |
| SK-53 | 83.889 | 86.667 | 5.978 | 6.489 | 59.222 | 7.011 | 0.161 | 171.667 | 0.979 |
| SK-54 | 104.779 | 130.111 | 6.600 | 4.411 | 45.333 | 6.656 | 0.156 | 187.889 | 1.003 |
| SK-55 | 65.556 | 68.667 | 6.878 | 5.544 | 76.556 | 6.622 | 0.179 | 177.333 | 0.856 |
| SK-56 | 84.444 | 84.333 | 7.256 | 6.556 | 100.556 | 6.533 | 0.175 | 180.000 | 0.963 |
| SK-57 | 80.889 | 78.778 | 7.111 | 6.478 | 61.111 | 6.600 | 0.165 | 193.444 | 0.838 |
| SK-58 | 81.111 | 83.889 | 6.911 | 6.444 | 38.222 | 6.611 | 0.185 | 190.222 | 0.966 |
| SK-59 | 82.778 | 92.556 | 8.222 | 8.200 | 113.556 | 7.856 | 0.142 | 190.111 | 0.863 |
| SK-60 | 80.778 | 87.778 | 6.189 | 6.844 | 49.000 | 6.211 | 0.152 | 177.000 | 0.913 |
| SK-61 | 69.333 | 84.556 | 6.622 | 6.511 | 43.222 | 6.400 | 0.150 | 195.000 | 0.988 |
| SK-62 | 84.778 | 79.556 | 6.133 | 6.267 | 68.778 | 6.600 | 0.204 | 183.889 | 0.869 |
| SK-63 | 82.111 | 77.222 | 6.700 | 6.578 | 66.889 | 6.667 | 0.155 | 173.444 | 1.214 |
| SK-64 | 84.333 | 81.667 | 7.033 | 6.544 | 26.222 | 6.667 | 0.188 | 177.000 | 0.918 |
| SK-65 | 71.667 | 71.556 | 6.767 | 6.356 | 28.667 | 6.733 | 0.182 | 183.222 | 1.084 |
| SK-66 | 79.556 | 80.889 | 6.356 | 5.667 | 55.333 | 6.600 | 0.164 | 161.889 | 0.863 |
| SK-67 | 80.333 | 87.889 | 7.822 | 7.711 | 45.556 | 6.533 | 0.143 | 159.333 | 0.831 |
| SK-68 | 79.556 | 79.000 | 7.444 | 6.822 | 54.333 | 6.200 | 0.138 | 160.222 | 0.977 |
| SK-69 | 84.556 | 93.222 | 6.922 | 6.611 | 71.889 | 6.789 | 0.142 | 150.778 | 0.954 |
| SK-70 | 82.111 | 74.778 | 5.211 | 7.000 | 36.111 | 6.400 | 0.208 | 166.111 | 0.831 |
| SK-71 | 90.222 | 196.333 | 6.100 | 6.311 | 86.333 | 6.767 | 0.210 | 182.333 | 1.161 |
| SK-72 | 84.778 | 80.000 | 7.067 | 6.611 | 65.000 | 6.278 | 0.142 | 172.222 | 0.888 |
| SK-73 | 83.000 | 83.667 | 6.833 | 7.089 | 40.111 | 6.411 | 0.144 | 173.556 | 0.962 |
| SK-74 | 86.889 | 81.111 | 7.478 | 6.867 | 81.444 | 6.489 | 0.141 | 186.222 | 0.820 |
| SK-75 | 80.889 | 80.333 | 7.211 | 6.700 | 43.222 | 6.278 | 0.158 | 182.111 | 0.879 |
| SK-76 | 81.111 | 85.111 | 7.256 | 7.067 | 55.111 | 6.244 | 0.146 | 182.222 | 0.813 |
| SK-77 | 75.667 | 77.444 | 7.233 | 6.733 | 21.889 | 6.644 | 0.148 | 176.667 | 0.952 |
| SK-78 | 100.778 | 127.667 | 5.167 | 6.911 | 66.111 | 6.389 | 0.152 | 197.333 | 0.968 |
| SK-79 | 89.778 | 83.667 | 6.600 | 6.444 | 50.667 | 6.689 | 0.166 | 191.444 | 0.839 |
| SK-80 | 82.222 | 72.111 | 7.089 | 6.578 | 63.889 | 6.544 | 0.155 | 180.000 | 0.956 |
| SK-81 | 82.222 | 86.556 | 6.656 | 6.900 | 117.556 | 7.922 | 0.154 | 178.778 | 0.951 |
| SK-82 | 88.667 | 88.000 | 6.533 | 6.656 | 82.333 | 6.778 | 0.212 | 187.111 | 0.824 |
| SK-83 | 81.667 | 121.222 | 5.611 | 6.422 | 126.778 | 6.922 | 0.122 | 167.222 | 0.822 |
| SK-84 | 77.111 | 78.444 | 7.389 | 6.789 | 77.444 | 6.522 | 0.194 | 147.000 | 0.817 |
| SK-85 | 94.444 | 216.333 | 8.344 | 8.356 | 124.667 | 7.756 | 0.259 | 167.111 | 0.943 |
| SK-86 | 82.222 | 79.667 | 7.344 | 6.611 | 107.778 | 6.267 | 0.203 | 155.889 | 0.933 |
| SK-87 | 86.222 | 81.000 | 7.478 | 6.611 | 56.889 | 7.089 | 0.171 | 171.778 | 0.821 |
| SK-88 | 87.778 | 87.556 | 6.344 | 6.822 | 72.444 | 6.922 | 0.153 | 187.667 | 0.862 |
| SK-89 | 87.778 | 121.333 | 6.156 | 3.633 | 62.222 | 6.111 | 0.122 | 178.889 | 0.861 |
| SK-90 | 102.778 | 116.556 | 5.133 | 4.333 | 37.111 | 6.333 | 0.162 | 177.556 | 0.922 |
| SK-91 | 87.111 | 81.556 | 7.389 | 7.311 | 44.000 | 7.544 | 0.165 | 191.111 | 0.866 |
| SK-92 | 83.778 | 77.111 | 7.267 | 6.600 | 64.667 | 6.789 | 0.172 | 187.444 | 0.831 |
| SK-93 | 87.778 | 98.000 | 6.556 | 6.478 | 42.778 | 5.856 | 0.144 | 189.222 | 0.986 |
| SK-94 | 77.333 | 77.556 | 6.267 | 6.667 | 116.333 | 6.567 | 0.161 | 183.889 | 1.054 |
| SK-95 | 76.889 | 78.667 | 7.289 | 6.200 | 39.111 | 6.600 | 0.145 | 200.444 | 0.870 |
| SK-96 | 81.333 | 84.333 | 7.611 | 6.267 | 58.444 | 6.811 | 0.146 | 191.333 | 0.816 |
| SK-97 | 81.667 | 84.556 | 7.067 | 6.433 | 51.556 | 6.611 | 0.157 | 177.889 | 0.891 |
| SK-98 | 77.889 | 81.556 | 6.978 | 6.333 | 87.222 | 6.589 | 0.180 | 178.667 | 0.858 |
| SK-99 | 90.556 | 87.556 | 7.511 | 6.711 | 38.000 | 6.500 | 0.159 | 187.667 | 0.846 |
| SK-100 | 77.556 | 83.778 | 7.411 | 6.133 | 88.333 | 6.722 | 0.165 | 167.333 | 0.879 |
| SK-101 | 98.000 | 115.556 | 8.289 | 6.978 | 69.778 | 5.100 | 0.144 | 166.667 | 0.977 |
| SK-102 | 83.889 | 84.111 | 6.589 | 6.356 | 50.444 | 6.633 | 0.134 | 163.444 | 0.863 |
| SK-103 | 84.556 | 78.778 | 7.300 | 6.167 | 88.556 | 6.600 | 0.140 | 158.111 | 1.100 |
| SK-104 | 82.556 | 79.000 | 7.144 | 6.722 | 51.111 | 7.311 | 0.154 | 169.333 | 0.862 |
| SK-105 | 96.444 | 126.889 | 6.811 | 3.767 | 87.667 | 7.156 | 0.155 | 187.222 | 0.828 |
| SK-106 | 94.444 | 103.222 | 6.422 | 5.556 | 62.000 | 6.456 | 0.172 | 175.222 | 0.890 |
| SK-107 | 76.778 | 80.556 | 6.644 | 6.256 | 120.444 | 6.756 | 0.158 | 176.667 | 0.948 |
| SK-108 | 79.111 | 87.778 | 7.267 | 6.333 | 88.444 | 6.667 | 0.145 | 186.667 | 0.850 |
| SK-109 | 78.111 | 84.111 | 7.289 | 6.289 | 82.333 | 6.611 | 0.176 | 184.444 | 0.874 |
| SK-110 | 81.889 | 85.778 | 7.489 | 6.344 | 36.222 | 6.767 | 0.145 | 181.444 | 0.977 |
| SK-111 | 120.556 | 135.444 | 5.689 | 5.733 | 91.667 | 6.800 | 0.145 | 176.889 | 0.916 |
| SK-112 | 118.000 | 130.444 | 5.311 | 3.467 | 59.778 | 7.233 | 0.143 | 186.222 | 0.847 |
| SK-113 | 81.667 | 80.222 | 6.433 | 6.133 | 86.889 | 7.722 | 0.138 | 188.333 | 0.836 |
| SK-114 | 119.444 | 137.333 | 5.500 | 2.667 | 77.667 | 6.444 | 0.225 | 180.778 | 0.849 |
| SK-115 | 105.222 | 135.444 | 6.644 | 4.022 | 193.889 | 6.344 | 0.221 | 176.444 | 0.923 |
| SK-116 | 96.000 | 108.667 | 7.522 | 6.111 | 95.778 | 8.233 | 0.176 | 184.889 | 0.890 |
| SK-117 | 94.889 | 132.778 | 5.544 | 3.056 | 94.667 | 7.633 | 0.147 | 178.222 | 0.846 |
| SK-118 | 81.000 | 73.111 | 7.433 | 6.456 | 71.111 | 6.911 | 0.133 | 189.000 | 0.857 |
| SK-119 | 120.333 | 146.889 | 5.900 | 4.411 | 61.111 | 5.033 | 0.207 | 189.889 | 0.916 |
| SK-120 | 94.556 | 123.444 | 5.567 | 2.511 | 80.111 | 7.178 | 0.124 | 198.667 | 0.853 |
| SK-121 | 116.222 | 133.556 | 5.867 | 4.544 | 60.778 | 6.811 | 0.145 | 179.222 | 0.821 |
| SK-122 | 90.000 | 77.556 | 7.022 | 6.144 | 78.778 | 6.533 | 0.149 | 188.889 | 0.862 |
| SK-123 | 69.778 | 76.889 | 7.111 | 5.622 | 16.667 | 6.389 | 0.127 | 190.444 | 1.332 |
| SK-124 | 77.333 | 87.667 | 6.989 | 6.067 | 51.222 | 6.800 | 0.155 | 179.889 | 1.126 |
| SK-125 | 82.889 | 98.556 | 6.456 | 6.044 | 48.444 | 6.800 | 0.154 | 167.556 | 0.852 |
| SK-126 | 106.778 | 113.111 | 5.778 | 5.189 | 63.333 | 6.889 | 0.175 | 159.444 | 0.936 |
| SK-127 | 78.222 | 81.111 | 7.033 | 6.333 | 36.111 | 6.489 | 0.165 | 158.111 | 0.864 |
| SK-128 | 108.667 | 129.000 | 6.778 | 3.867 | 78.333 | 6.033 | 0.196 | 168.889 | 0.994 |
| SK-129 | 83.111 | 81.556 | 6.567 | 5.967 | 38.778 | 5.989 | 0.145 | 180.222 | 0.979 |
| SK-130 | 82.889 | 75.889 | 7.556 | 6.578 | 59.556 | 7.078 | 0.145 | 169.333 | 0.864 |
| SK-131 | 87.444 | 77.556 | 7.244 | 6.133 | 69.889 | 7.167 | 0.142 | 185.444 | 0.894 |
| SK-132 | 83.333 | 82.444 | 6.822 | 6.011 | 143.444 | 6.578 | 0.137 | 186.556 | 0.928 |
| SK-133 | 93.556 | 98.000 | 6.133 | 5.489 | 73.667 | 5.844 | 0.119 | 178.778 | 0.852 |
| SK-134 | 82.556 | 121.333 | 5.344 | 2.644 | 137.556 | 7.056 | 0.154 | 178.444 | 0.913 |
| SK-135 | 87.889 | 79.222 | 6.333 | 6.111 | 66.000 | 6.589 | 0.123 | 192.111 | 0.921 |
| SK-136 | 106.889 | 115.444 | 5.422 | 4.389 | 169.444 | 6.533 | 0.144 | 188.111 | 0.961 |
| SK-137 | 100.667 | 112.667 | 4.900 | 4.322 | 84.000 | 7.611 | 0.158 | 188.556 | 0.954 |
| SK-138 | 108.778 | 123.111 | 6.467 | 4.100 | 79.667 | 5.700 | 0.137 | 182.889 | 0.864 |
| SK-139 | 84.778 | 87.444 | 7.322 | 7.089 | 40.556 | 6.489 | 0.125 | 196.333 | 0.978 |
| SK-140 | 79.556 | 76.889 | 6.044 | 5.900 | 58.222 | 6.933 | 0.139 | 186.778 | 0.853 |
| SK-141 | 94.444 | 128.667 | 5.644 | 7.033 | 33.000 | 5.511 | 0.170 | 175.444 | 0.924 |
| SK-142 | 92.556 | 150.222 | 5.700 | 3.067 | 118.333 | 6.056 | 0.135 | 172.222 | 0.739 |
| SK-143 | 112.111 | 117.889 | 6.678 | 5.578 | 62.333 | 6.567 | 0.126 | 162.000 | 0.870 |
| SK-144 | 81.556 | 90.000 | 6.167 | 6.300 | 30.000 | 6.844 | 0.139 | 158.556 | 1.123 |
| SK-145 | 87.333 | 81.556 | 6.567 | 6.211 | 72.000 | 6.189 | 0.167 | 160.667 | 0.862 |
| SK-146 | 78.889 | 80.111 | 6.911 | 5.989 | 26.000 | 6.800 | 0.141 | 154.000 | 1.197 |
| SK-147 | 80.333 | 76.222 | 7.500 | 6.611 | 40.000 | 6.667 | 0.137 | 169.222 | 0.976 |
| SK-148 | 80.111 | 71.556 | 7.122 | 6.300 | 50.556 | 6.344 | 0.132 | 183.444 | 1.139 |
| SK-149 | 84.111 | 76.889 | 7.367 | 6.311 | 71.556 | 6.767 | 0.123 | 176.889 | 0.857 |
| SK-150 | 78.667 | 82.556 | 7.878 | 6.544 | 48.333 | 7.511 | 0.156 | 172.778 | 0.922 |
| SK-151 | 87.778 | 78.000 | 6.656 | 6.133 | 58.333 | 6.656 | 0.152 | 186.889 | 0.857 |
| SK-152 | 80.778 | 79.778 | 6.433 | 6.267 | 40.333 | 6.611 | 0.184 | 183.556 | 0.912 |
| SK-153 | 81.556 | 86.000 | 6.611 | 6.089 | 64.333 | 6.656 | 0.111 | 184.667 | 0.929 |
| SK-154 | 79.333 | 83.111 | 6.622 | 6.256 | 64.889 | 6.600 | 0.158 | 176.778 | 0.996 |
| SK-155 | 85.556 | 75.889 | 6.744 | 6.400 | 86.444 | 6.333 | 0.201 | 195.667 | 0.910 |
| SK-156 | 158.889 | 202.222 | 8.122 | 7.167 | 86.111 | 7.744 | 0.136 | 186.333 | 0.860 |
| SK-157 | 78.778 | 97.333 | 7.200 | 6.000 | 47.444 | 6.511 | 0.141 | 179.889 | 0.914 |
| SK-158 | 86.667 | 91.444 | 5.956 | 5.189 | 49.778 | 6.600 | 0.143 | 176.111 | 0.829 |
| SK-159 | 91.333 | 76.556 | 7.233 | 6.589 | 29.556 | 6.667 | 0.139 | 183.111 | 0.822 |
| SK-160 | 82.000 | 87.444 | 6.178 | 6.800 | 51.889 | 6.756 | 0.123 | 174.444 | 1.034 |
| SK-161 | 99.444 | 193.333 | 7.944 | 8.189 | 90.000 | 7.333 | 0.134 | 175.889 | 0.870 |
| SK-162 | 83.222 | 95.667 | 6.456 | 6.500 | 38.778 | 6.189 | 0.125 | 180.889 | 0.924 |
| SK-163 | 93.111 | 95.222 | 6.389 | 6.344 | 44.333 | 6.400 | 0.116 | 184.556 | 0.949 |
| SK-164 | 75.444 | 77.222 | 6.489 | 6.656 | 24.333 | 6.822 | 0.173 | 174.333 | 0.946 |
| SK-165 | 73.889 | 81.333 | 6.778 | 6.533 | 53.444 | 7.100 | 0.135 | 182.778 | 1.136 |
| SK-166 | 78.000 | 80.667 | 6.867 | 6.722 | 27.222 | 6.700 | 14.220 | 184.333 | 1.231 |
| SK-167 | 104.222 | 110.111 | 7.178 | 5.811 | 40.333 | 6.456 | 0.126 | 172.667 | 1.070 |
| SK-168 | 82.000 | 81.111 | 7.244 | 6.700 | 49.333 | 6.500 | 0.136 | 158.333 | 1.047 |
| SK-169 | 84.222 | 79.222 | 6.511 | 6.778 | 29.222 | 6.767 | 0.126 | 153.556 | 1.201 |
| SK-170 | 82.667 | 81.444 | 6.844 | 6.556 | 56.222 | 6.867 | 0.170 | 160.111 | 0.851 |
| SK-171 | 99.778 | 97.222 | 6.789 | 6.422 | 35.000 | 6.389 | 0.166 | 170.333 | 1.140 |
| SK-172 | 87.667 | 87.111 | 7.856 | 7.933 | 53.444 | 8.644 | 0.157 | 181.111 | 0.831 |
| SK-173 | 80.444 | 74.889 | 7.400 | 6.678 | 51.444 | 6.889 | 0.149 | 167.667 | 1.037 |
| SK-174 | 70.333 | 65.889 | 6.756 | 6.489 | 31.333 | 7.033 | 0.166 | 186.222 | 1.043 |
| SK-175 | 76.222 | 217.000 | 5.356 | 5.944 | 87.222 | 6.983 | 0.140 | 190.556 | 0.856 |
| SK-176 | 81.222 | 86.556 | 7.289 | 6.589 | 70.333 | 6.211 | 0.143 | 182.000 | 0.844 |
| SK-177 | 86.333 | 81.556 | 6.633 | 5.922 | 69.111 | 6.311 | 0.162 | 200.000 | 0.982 |
| SK-178 | 89.779 | 81.333 | 5.900 | 4.522 | 40.000 | 6.744 | 0.153 | 190.556 | 0.970 |
| SK-179 | 76.111 | 76.222 | 6.889 | 6.244 | 76.778 | 6.700 | 0.134 | 177.222 | 0.842 |
| SK-180 | 81.222 | 87.000 | 6.367 | 6.467 | 56.333 | 6.744 | 0.169 | 179.111 | 0.876 |
| SK-181 | 79.778 | 82.111 | 6.700 | 6.500 | 34.778 | 6.211 | 0.163 | 184.778 | 0.754 |
| SK-182 | 77.556 | 77.222 | 7.656 | 6.844 | 62.111 | 6.389 | 0.190 | 162.333 | 0.822 |
| SK-183 | 85.333 | 143.333 | 7.656 | 6.478 | 71.000 | 6.644 | 0.139 | 160.111 | 0.819 |
| SK-184 | 78.889 | 160.667 | 7.178 | 6.200 | 47.333 | 6.211 | 0.141 | 162.444 | 0.913 |
| SK-185 | 88.889 | 172.778 | 8.178 | 7.233 | 53.444 | 7.311 | 0.158 | 152.778 | 0.873 |
| SK-186 | 82.111 | 79.111 | 7.422 | 6.233 | 38.333 | 6.400 | 0.182 | 168.667 | 0.852 |
